# Two-Dimensional Carbon-Based Electrochemical Sensors for Pesticide Detection: Recent Advances and Environmental Monitoring Applications

**DOI:** 10.3390/bios16010062

**Published:** 2026-01-14

**Authors:** K. Imran, Al Amin, Gajapaneni Venkata Prasad, Y. Veera Manohara Reddy, Lestari Intan Gita, Jeyaraj Wilson, Tae Hyun Kim

**Affiliations:** 1 Department of Chemistry, Madanapalle Institute of Technology & Science (MITS), Deemed to be University, Madanapalle 517325, India; imransvu@gmail.com; 2 Department of Chemistry, Soonchunhyang University, Asan 31538, Republic of Korea; 3 Department of Chemistry, Presidency University, Bengaluru 560064, India; 4 Department of Chemistry, Sri Venkateswara College, University of Delhi, New Delhi 110021, India; 5 Polymer Electronics Lab, Department of Bioelectronics and Biosensors, Alagappa University, Karaikudi 630003, India

**Keywords:** 2D carbon nanomaterials, sensor fabrication, food safety, pesticides, environmental monitoring

## Abstract

Pesticides have been widely applied in agricultural practices over the past decades to protect crops from pests and other harmful organisms. However, their extensive use results in the contamination of soil, water, and agricultural products, posing significant risks to human and environmental health. Exposure to pesticides can lead to skin irritation, respiratory disorders, and various chronic health problems. Moreover, pesticides frequently enter surface water bodies such as rivers and lakes through agricultural runoff and leaching processes. Therefore, developing effective analytical methods for the rapid and sensitive detection of pesticides in food and water is of great importance. Electrochemical sensing techniques have shown remarkable progress in pesticide analysis due to their high sensitivity, simplicity, and potential for on-site monitoring. Two-dimensional (2D) carbon nanomaterials have emerged as efficient electrocatalysts for the precise and selective detection of pesticides, owing to their large surface area, excellent electrical conductivity, and unique structural features. In this review, we summarize recent advancements in the electrochemical detection of pesticides using 2D carbon-based materials. Comprehensive information on electrode fabrication, sensing mechanisms, analytical performance—including sensing range and limit of detection—and the versatility of 2D carbon composites for pesticide detection is provided. Challenges and future perspectives in developing highly sensitive and selective electrochemical sensing platforms are also discussed, highlighting their potential for simultaneous pesticide monitoring in food and environmental samples. Carbon-based electrochemical sensors have been the subject of many investigations, but their practical application in actual environmental and food samples is still restricted because of matrix effects, operational instability, and repeatability issues. In order to close the gap between laboratory research and real-world applications, this review critically examines sensor performance in real-sample conditions and offers innovative approaches for in situ pesticide monitoring.

## 1. Introduction

**Figure 1:**
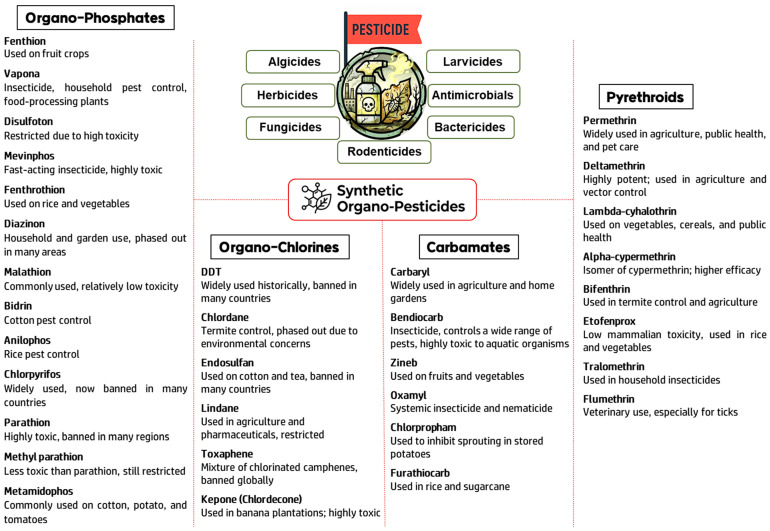
A general overview of the classification of some major synthetic organo-pesticides and their typical applications.

The accelerating growth of the global population leads to significant demand for food supply, making it difficult to achieve greater yields in agriculture due to problems such as poor soil health, lack of fertilizers, unpredictable weather conditions, irrigation issues, crop infections, harmful parasites, and plant-eating pests. One notable advancement is the emergence of precision agriculture or smart agriculture, which is garnering significant attention for its ability to optimize resource use while enhancing productivity. As a critical driver of increasing agricultural production, pesticides are widely applied to crops across the globe due to their effectiveness in controlling plant pests, thereby minimizing the need for manual labour [1]. Pesticides encompass various substances, including herbicides used to terminate weeds and other undesirable vegetation; insecticides for controlling a broad array of insect species; fungicides used to prevent the growth of moulds and mildew; disinfectants to curb the spread of harmful bacteria; and rodenticides for controlling the population of mice and rats [2]. The toxic nature of pesticides affects not only the intended pest species but also disrupts ecosystems by damaging the habitats of beneficial organisms. Additionally, many pesticides exhibit environmental persistence, allowing them to remain active for prolonged periods. These properties trigger their accumulation in the tissues of organisms and subsequent transfer through trophic levels, leading to bioaccumulation and biomagnification. Furthermore, pesticides can significantly affect water purity and quality by dispersing into adjacent ecosystems, leading to the contamination of aquatic life and fertile soil. Prolonged and intensive use of pesticides can substantially heighten environmental risks by accelerating resistance development in target pests and promoting the emergence of secondary pest infestations [3]. India ranks fourth in agrochemical production, yet its pesticide usage remains relatively low on a per-hectare basis compared to countries such as Japan, South Korea, and the United States. Soil is the main reservoir for pesticides applied in agricultural practices. While only around 10% of these chemicals effectively reach their intended targets, a significant portion, ranging from 20% to 70%, along with their breakdown products, end up in the soil, where they can persist as long-lasting residues. Based on their predominant chemical constituents, pesticides are systematically classified into organochlorines, organophosphates, and carbamates [4,5,6]. Based on the exposure (amount and location), certain pesticides are toxic to other animals and humans if they enter our food chain through sources such as air pollution, groundwater, food storage, or harvest. The primary cause of illnesses that seriously impair our health is contaminated food [2]. The recent investigation suggested that edible plants are contaminated by approximately 30% with pesticide residues [7]. Regulation (EC) No 396/2005 establishes maximum residue levels (MRLs) of pesticide residues in food and feed of plant and animal origin intended for human or animal consumption at 0.01 ppm [1]. The high exposure of plant tissues to pesticides can lead to a phytotoxic effect, oxidative damage and alteration of enzyme systems impacting the physiological activities of plants [8]. The prolonged exposure to pesticides in humans leads to the development of neurological diseases due to their inhibitory action on acetylcholinesterase (AChE) [9]. Nerve damage in both humans and animals causes neurological problems that can affect mobility, sensation, gland secretion, or organ function. Moreover, the affected nerves may cause other serious health problems. Several environmental protection groups have classified pesticides as a priority hazardous substance. A few of the toxic pesticide components are shown in Figure 1. The routine techniques, including mass spectrometry, liquid–gas chromatography, Raman, fluorescence spectroscopy, etc., are frequently used to detect pesticides [10,11,12]. The speed and accessibility of analysis techniques, high selectivity with sensitivity, interfering molecules, and high-cost levels are only a few of the numerous obstacles and demands that still need to be addressed. Electrochemical sensing mechanisms are crucial in many applications because they employ a range of transduction strategies to detect analytes with high sensitivity and selectivity. The primary techniques are impedimetric, potentiometric, amperometric, and transistor-based sensing; each offers unique advantages for certain applications [2]. An interesting area of research in electrochemical detection is electrochemical (EC) sensors, a potential alternative sensor platform for detecting chemical pollutants, pesticides, etc., [13]. These sensors can be seamlessly integrated into compact, portable devices for rapid and real-time analysis in various application areas, including agro-environmental monitoring, food quality, and safety [14]. When optimally engineered for pesticide detection, EC sensors exhibit exceptional sensitivity and selectivity, even in complex sample matrices. In electrochemical detection, functional materials can be used to modify the working electrode, enabling it to detect the target pesticide with high sensitivity and selectivity while responding much more quickly. The EC sensors work on the following fundamental mechanisms: electrocatalysis, the signal transduction process, and the use of functional materials [15]. These sensors convert chemical or biological interactions into electrical signals via transducers, which can be electrodes or other materials. The performance of these sensors is influenced by the immobilization of biomolecules and the electrode’s surface properties. Moreover, enhanced electron-transfer processes increase sensitivity and specificity by boosting electron transfer mechanisms. Electrocatalytic efficiency is strongly influenced by concentration and kinetic factors. Additionally, the use of advanced materials like nanomaterials and metal–organic frameworks improves sensor performance and contributes to stability and selectivity under varied conditions by increasing mass transport and surface area [16]. Even though electrochemical sensors have a lot of potential, concerns like stability in many environmental scenarios and the requirement for more breakthroughs in sensor design continue to be crucial for this field’s future developments.

Several sensing techniques are used in the electrochemical detection of pesticides utilizing 2D carbon-based materials, depending on the electrode architecture and pesticide class. Analyte accumulation on graphene or g-C_3_N_4_ surfaces is facilitated by strong π–π interactions and hydrogen bonding, which are characteristic adsorption-controlled processes for organophosphates and carbamates. In sensors with metal nanoparticles or heterostructures, electrocatalytic mechanisms predominate, allowing for higher sensitivity and enhanced redox reactions. Inhibition-based sensing, which is especially pertinent to organophosphate pesticides, makes use of the reduction in enzyme activity, such as acetylcholinesterase, to produce detectable changes in current. Affinity-based techniques that use aptamer-functionalized electrodes or molecularly imprinted polymers also provide great selectivity toward certain pesticide compounds, which makes them appropriate for complicated sample matrices.

**Figure 2:**
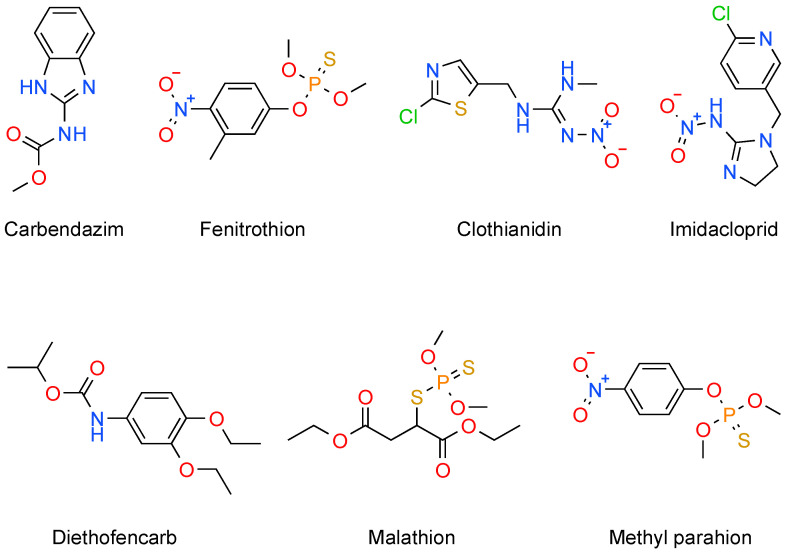
Chemical structures of several hazardous pesticides.

While a lot of work has gone into creating extremely sensitive electrochemical sensors for pesticide detection, sensitivity by itself does not guarantee dependable operation in real-world scenarios. Complex matrices comprising organic matter, inorganic ions, and coexisting pollutants can significantly affect electrochemical responses in real environmental and food samples, frequently resulting in signal suppression, amplification, or electrode fouling. The translation of laboratory-scale sensor performance to practical applications is severely hampered by these matrix effects. Therefore, a vital understanding of matrix effects and how they affect electrochemical sensing is necessary for the logical design of sensors that can detect pesticides in complex samples with accuracy and repeatability. The following section examines the causes, implications, and mitigation techniques for matrix effects in electrochemical pesticide sensing. In this context, the present review focused on the electrochemical detection of several kinds of pesticides, whose chemical structures are summarized in Figure 2.

## 2. Sensor Design Strategies Based on Pesticide Chemical Classes

### 2.1. Organophosphates

Among the most popular insecticides, organophosphate pesticides are distinguished by the presence of a functional group containing phosphorus. Their great capacity to inhibit acetylcholinesterase (AChE) via phosphorylating the enzyme’s active site accounts for their principal toxicological and electrochemical relevance [17]. Many electrochemical sensing methods are based on this inhibitory mechanism. Since organophosphates usually show little direct redox activity from an electrochemical perspective, enzyme inhibition-based methods are frequently used to detect them indirectly. In such systems, the decrease in enzyme activity (for example, reduced thiocholine oxidation) is electrochemically measured and associated with the concentration of organophosphate. Surface-modified electrodes, such as those with nanostructured carbon materials, metal nanoparticles, or conducting polymers, are commonly used to improve sensitivity and selectivity [18].

### 2.2. Carbamates

A class of nitrogen-containing chemicals called carbamate insecticides is frequently employed in the management of agricultural pests. They differ from organophosphate pesticides in that their main biochemical effect is the reversible suppression of acetylcholinesterase via carbamylation of the enzyme active site [18]. From an electrochemical perspective, carbamates frequently show substantial intrinsic redox activity at electrode surfaces instead of adsorption-dominated behaviour. As a result, the design of electrochemical sensors for carbamate detection places a strong emphasis on the selection of suitable electrode materials, surface functionalization to increase adsorption efficiency, and signal amplification techniques utilizing nanostructured materials to boost analytical performance and sensitivity.

### 2.3. Organochlorine Pesticides

Organochlorine pesticides are distinguished by a high level of chlorination, which provides great hydrophobicity and chemical durability. Because of their low inherent electrochemical redox activity, their detection is mostly determined by adsorption-controlled processes and hydrophobic interactions on the electrode surface [19]. In order to improve adsorption efficiency and analyte preconcentration, electrochemical sensor design strategies prioritize surface-modified electrodes that incorporate functional nanomaterials. Furthermore, tailored sensing interfaces and affinity-based recognition elements are used to enhance analytical performance and selectivity in complicated environmental matrices.

### 2.4. Pyrethroid Pesticides

Pyrethroid pesticides are synthetic derivatives of natural pyrethrins that are commonly utilized due to their great insecticidal efficiency and minimal mammalian toxicity. Pyrethroids often show limited direct redox activity electrochemically and are mostly identified by indirect sensing mechanisms or adsorption-controlled procedures [20]. In order to improve adsorption efficiency and signal transduction, surface-modified electrodes, functional nanomaterials, and affinity-based interfaces are prioritized in electrochemical sensor design for pyrethroid detection. To increase sensitivity and selectivity, customized sensing methods are frequently used, such as molecular recognition layers and electrodes modified with nanocomposite [20].

## 3. Matrix Effect in Electrochemical Detection of Pesticides

Matrix effect is a significant challenge in electrochemical analysis, as it is caused by the presence of interfering compounds in real matrices that can either suppress or enhance the electrochemical signal. In electrochemical pesticide sensing, the analytical response can be significantly influenced by coexisting species present in environmental matrices, such as soil, food extracts, wastewater, agricultural runoff water, and river and lake water. The signal accuracy is altered by interfering substances, which produce overlapping redox signals, alter electron transfer kinetics, compete for adsorption sites and foul the electrode surface. The inorganic ions Ni^2+^, Co^2+^, Fe^3+^, and Cu^2+^ can primarily form complexes with certain pesticides [21]. Nitroaromatic substances, which closely resemble the electrochemical behaviour of nitro-containing pesticides, can also interfere with detection. Phenolic compounds mainly interfere with carbon-based electrodes through π-π adsorption, thereby limiting the availability of active sites [22]. Some biomolecules, such as dopamine, uric acid and ascorbic acid, can induce surface fouling or contribute substantial background currents, while humic substances, surfactants, and saccharides (sucrose and glucose, etc.) block catalytic pores and hamper diffusion. Hence, a selective electrochemical sensing platform requires minimizing the matrix-derived artefacts either through an antifouling coating or preferential adsorption of the catalyst, or proper host–guest recognition. In this context, researchers have concentrated on the synthesis and practical applications of 2D-carbon derived materials, which have been applied in the analysis of various real samples.

## 4. Overview of 2D Nanomaterials Towards Sensing of Pesticides

### 4.1. Graphene

Graphene is a vital two-dimensional, sp^2^ hybridized polycyclic aromatic carbon nanomaterial that appears to be a skeletal honeycomb and can be further functionalized or modified better with various functional groups (-OH, -O-, -COOH and -NH_2_, etc.), polymers, metal, metal oxide and other substances. Graphene exhibits low magnetic properties due to short-term electron–electron interactions and long-term coulomb interactions, making it a low magnetic noise, having high carrier mobility and high surface sensitivity [23,24]. Two-dimensional graphene (2D-G) has high conductivity in terms of both thermal and electrical aspects due to its resistance to moisture and high electron mobility. The 2D-G conductivity and optical transparency have led to its application as a transparent conductive layer in advanced photonic devices [25]. The 2D-G exhibits major properties that position it as a key material for transformative innovations in various scientific domains. This makes 2D-G a crucial material for application in batteries and cells as anodes, and solar panels, supercapacitors, fuel cells, anticorrosion coatings, DNA sequencing, drug delivery, biosensors, and as a significant physical surface support for desired biomolecules to transport into the solution media in electrochemical sensor applications. The 2D-G, characterized by its highly conjugated π-electron network and an exceptional specific surface approaching nearly 2600 m^2^ g^−1^, demonstrates superior adsorption efficacy towards organic molecules, owing to its electronic structure and expansive interfacial domain [26,27,28,29]. These unique, complex, and multifaceted properties of 2D-G, along with its high surface-to-volume ratio and porosity, are poised to advance future developments by enhancing the interaction between the analyte and the electrode surface, thereby promoting improved sensitivity and detection limits, highlighting the critical role of the electrode in real-time detection and monitoring of pesticides [30,31,32,33,34,35,36].

### 4.2. Derivatives of 2D-G

The enhanced structural versatility of graphene-based derivatives accelerates fast electron transfer kinetics, efficient mass transport of analytes, and permits selective functionalization with suitable moieties, thereby enabling selective and sensitive analysis of selected analytes in electrochemical sensing [37]. The popular 2D-G derivatives, such as graphene oxide, reduced graphene oxide, and carbon nanowalls, are significant functionalized materials. Two-dimensional graphene oxide (2D-GO) material is composed of oxygen-rich functional groups, which exhibit varied electronic character, chemical stability, lower conductivity, and high reactivity compared to 2D-G. The 2D-GO is prepared by the oxidation of graphene, which produces a dispersible aqueous solution. The plane structure of 2D-GO, proven to be primarily composed of epoxy (-O-) and hydroxyl (-OH) groups, is very appropriate for non-carbon atom doping or polymer bonding applications, making it accessible as an electrode modification material [36]. The two-dimensional reduced graphene oxide (2D-rGO) is achieved by decreasing or reducing the oxygen functional groups from 2D-GO. By approaching or restoring the sp^2^ carbon network of 2D-G, 2D-rGO enhances its interaction capabilities with analytes through various underlying mechanisms such as π-π interactions, hydrogen bonding, and electrostatic forces [38,39,40]. The 2D-rGO goes a step forward compared with 2D-GO, because of its different flexibilities, simple preparation method, ease of dispersion in solvents, electrical performance control and other physical properties like exceptional mechanical performance, electrical charge transfer ability, biological compatibility, and superior light transmittance, making it a potential candidate in biosensing and other sensor materials [41,42]. The further improvement of sensing or redox performance on the electrode surface can be greatly achieved by modifying 2D-rGO with suitable conducting materials [43].

### 4.3. Graphitic Carbon Nitride

Two-dimensional graphitic carbon nitride (2D-g-C_3_N_4_) sp^2^-hybridized carbon and nitrogen material, resembling a graphene-like polymeric structure, exhibits distinctive physical and chemical properties [44]. The material exhibits remarkable performance due to several inherent characteristics. It can be synthesized through environmentally sustainable and economical routes using precursors such as melamine, urea, or thiourea via a straightforward thermal polymerization process. Its tri-s-triazine ring structure and high degree of polycondensation impart exceptional physicochemical stability. The material also shows great potential in energy generation and storage applications, along with distinct fluorescence behaviour. Possessing a bandgap of about 2.7 eV, it effectively absorbs blue light with wavelengths shorter than 475 nm. Its outstanding optical properties contribute to excellent photo and electrocatalytic activity. Moreover, the electronic structure can be finely tuned, enabling modulation of charge carrier distribution owing to its intrinsic polarity and semiconducting nature. Finally, the porous architecture enhances its surface reactivity, making it a highly promising candidate for environmental remediation applications [45,46,47,48,49,50]. The foremost benefit of 2D-g-C_3_N_4_ over graphene is its bandgap, which features outstanding capabilities in absorbing light, thereby enhancing its potential in water oxidation and reduction processes, photocatalysis, optoelectronics, and nano-sensing [51]. It has attracted considerable scholarly attention in drug delivery, tissue engineering, and wound dressing and is a very promising material in cancer predictions, therapy because of its nontoxicity, biocompatibility, and its capability to penetrate the natural tissues [52]. The morphology and electrochemical sensing properties of 2D-g-C_3_N_4_ are significantly improved in terms of electronic states, creating new active sites, compressed band structure and elevating charge transport rate by the addition of non-metal (nitrogen, sulphur, oxygen, carbon, and phosphorus), monodoping with germanium, metal, metal oxides, conductive nano carbon substances (like graphene and carbon nanotube) and MXene (transition metal carbides, nitrides) substances [53,54,55,56,57,58,59,60]. On the other hand, owing to the high surface area and porous nature of 2D-g-C_3_N_4_ nanosheets, it is highly appropriate for environmental stressor (pesticide residues) sensing applications.

### 4.4. Graphdiyne

Li et al. prepared thin films of two-dimensional graphdiyne (2D-GDY) through a cross-coupling reaction, which has become a prominent member of 2D carbon-based materials [61]. The 2D-GDY has a unique pattern of linear diacetylene linkages between benzene rings, creating spatial variations in charge density and enabling enhanced electron mobility, an extended π-system, and a consistent pore architecture that enables efficient mass transport across both in-plane and out-of-plane directions [62,63,64]. Diverging from 2D-G, the 2D-GDY lattice predominantly exhibits a finite band gap, imparting semiconducting electronic properties. Furthermore, unlike the brittle fracture behaviour commonly associated with 2D-G, 2D-GDY nanosheets demonstrate polymer-like stretchability, offering enhanced mechanical flexibility and low density [65,66]. Owing to the intrinsic properties of its sp-hybridized carbon atoms, along with the highly reactive C-C triple bonds and fully conjugated architecture, 2D-GDY demonstrates a pronounced capacity for electronic doping and chemical modifications. This opens avenues in scientific and biomedical fields, including optical properties, bio-imaging, sensing technology, targeted obtained drug delivery, cancer therapy and antimicrobial applications. The 2D-GDY exhibits a high extinction coefficient across broad wave lengths and strong NIR absorption with a large surface area, resulting in outstanding photo thermal conversion efficiency [67]. The 2D-GDY is actively involved in removal and separation technology, such as desalination or water treatment, gas separation and storage, organic separation, and heavy metal removal through their high pore density, π-conjugation, polarity and large surface area [68,69,70,71,72]. The planar and Dirac cone structure of 2D-GDY exhibits high carrier mobility, and surface area represents the enhanced charge transfer and surface interactions on modified electrodes, facilitating the enrichment of target analytes during electrochemical detection. In addition, 2D-GDY has a tunable bandgap, can be functionalized by hetero atom or groups to get 2D-GDY functionalized composites, which are potentially used as electrode materials for enabling outstanding conductivity and enhanced electrochemical reactivity with numerous active sites [73,74,75,76].

In electrochemical sensing mechanisms, 2D-G and 2D-g-C_3_N_4_ play complementary but distinct roles. The 2D-G derivatives exhibit outstanding electrical conductivity, a large electroactive surface area, and fast electron-transfer kinetics. Hence, sharp voltammetric peaks, high amperometric currents, low charge-transfer resistance, and ultralow detection limits could be achieved. However, their sensitivity and adsorption are relatively limited, and these shortcomings can be addressed through functionalization strategies such as 2D-GO/2D-rGO modification, defect engineering, or decoration with metal nanoparticles. Even after functionalization, the graphene derivative retains metal-like conductivity and faster heterogeneous electron-transfer kinetics, leading to higher peak currents, sharper and better-resolved voltammetric peaks, and lower detection limits for electroactive pesticides (organophosphates, nitro-pesticides, carbamates). In contrast, functionalized 2D-g-C_3_N_4_ shows improved adsorption and selectivity due to its N-rich surface. But its semiconducting framework limits charge transport, resulting in comparatively broader and lower-intensity peaks. Therefore, 2D-G functionalized composites outperform 2D-g-C_3_N_4_-based ones in peak quality and sensitivity, while 2D-g-C_3_N_4_ is better used as a complementary component to enhance selectivity rather than as the primary peak-generating material.

## 5. Synthesis of 2D Carbon-Based Materials

### 5.1. The 2D-GO

The 2D-GO is one of the most widely studied derivatives of graphite oxide because of its adjustable properties, various functionalities and scalable synthesis. The 2D-GO is generally synthesized from the graphite through a chemical oxidation process, which introduces high-density oxygen-containing functional groups, for example, hydroxyl group, carboxylic group and epoxy group in the graphene. Because of these, it enhances the dispersibility in water and also provides active sites for further modification. For these reasons, GO is often used as a precursor to graphene-based nanocomposites. The most widely used path for synthesizing GO is the Hummers’ method, first established in 1958. This method uses potassium permanganate (KMnO_4_) and sodium nitrate (NaNO_3_) in a strong sulfuric acid (H_2_SO_4_) medium for the conversion of graphite into GO [77]. It became the benchmark method owing to its relatively reduced reaction time and higher reproducibility compared to earlier methods. However, the traditional Hummers’ process generates hazardous nitrogen oxides and manganese-containing waste as a by-product, which raises considerable environmental and safety concerns [78]. To mitigate these limitations, numerous modified Hummers’ methods have been proposed. For example, Marcano et al. (2010) eliminated NaNO_3_ from the reaction and introduced a H_2_SO_4_/H_3_PO_4_ mixture, which increased oxidation efficiency, yielded higher quality 2D-GO and lowered toxic emissions. Such modifications not only enhanced safety but also facilitated more environmentally friendly large-scale synthesis [78]. Recently, investigations have increasingly focused on eco-friendly oxidation routes, utilizing mild oxidants and greener solvents to reduce hazardous waste. For example, hydrogen peroxide (H_2_O_2_) has been successfully applied as the only oxidizing agent in a dilute H_2_SO_4_ system, with the acid acting primarily as a medium and reusable control agent. These green synthesis techniques significantly minimize the consumption of harsh chemicals yet maintaining efficiency, making 2D-GO production safer, quicker, and more viable for large-scale and biomedical purposes [79]. Therefore, 2D-GO synthesis has progressed from the initial chemical oxidation routes to contemporary, environmentally friendly approaches. Although the Hummers’ method and its modification remain the most widely utilized, continued efforts to develop safer, sustainable, rapid and large-scale methods demonstrate the advancement in this field.

### 5.2. The 2D-rGO

The 2D-rGO is typically produced from 2D-GO via processes that remove oxygen-containing functional groups, which helps to restore the conjugated sp^2^ carbon lattice and electrical conductivity. Among the current strategies, chemical, thermal, and electrochemical reduction are the most studied approaches. Every technique offers unique benefits and challenges depending on its intended use.

#### 5.2.1. Chemical Reduction

Chemical reduction is the most employed method for synthesizing 2D-rGO, in which GO dispersions are processed with reducing agents that eliminate oxygenated functional groups and partially recover the conjugated sp^2^ carbon network. During this process, the GO powder is usually suspended in water or another solvent to create a homogeneous suspension, followed by the gradual addition of the reducing agent under agitation, heating, or controlled pH conditions to promote oxygen removal. The reduced product is then obtained by centrifugation, rinsed several times to remove residual reagents, and dried for subsequent use. Hydrazine hydrate was one of the initial reductants utilized, enabling efficient elimination of oxygen groups and producing conductive graphene-like nanosheets. However, its high toxicity, instability, and environmental risks significantly limit its large-scale production [80]. Sodium borohydride (NaBH_4_) is another powerful reductant that ensures efficient reduction, yielding rGO with enhanced conductivity. The process generally involves dispersing GO in an aqueous medium, subsequently, the dropwise addition of NaBH_4_ under continuous agitation at room temperature or moderate temperatures. Although effective, NaBH_4_ is prone to instability and rapid reaction, limiting its feasibility for industrial scale [81]. Greener alternatives have been designed to replace hazardous chemicals. For example, ascorbic acid (vitamin C) has been identified to be an ideal replacement due to its low toxicity, natural availability, and reducing power. Fernández-Merino et al. (2010) demonstrated that the use of vitamin C for the reduction in GO dispersions produces high-quality rGO with conductivity nearly equivalent to hydrazine-treated samples [82]. In 2015, Abdolhosseinzadeh et al. introduced a rapid and scalable vitamin C-based method in which GO is dispersed in an aqueous medium, incorporating vitamin C as the reductant, and the mixture is warmed under mild thermal conditions to generate rGO for large-scale applications [83]. Other mild reductants have also been investigated. Hydroquinone has served as a facile reductant for the conversion of GO into rGO, usually by mixing hydroquinone with aqueous GO dispersions under controlled pH and thermal conditions, leading to conductive graphene nanosheets [84]. In addition, gaseous hydrogen reduction performed at high temperatures following the thermal expansion of GO provides a route to high-quality graphene sheets with controllable thickness, although it requires specific high-temperature environments [85].

#### 5.2.2. Thermal Reduction

Thermal reduction is one of the easiest approaches to produce rGO from GO. The process involves thermal treatment of GO at elevated temperatures under inert gas (Ar, N_2_) or reducing (H_2_/Ar) environments, leading to the decomposition of oxygen-containing moieties (hydroxyl, epoxy, and carboxyl) into gaseous species such as CO_2_, CO, and H_2_O. Such annealing restores the extended sp^2^ carbon lattice and significantly improves the electrical conductivity of the obtained material. Larciprete et al. (2011) contributed the mechanical insights into thermal reduction, highlighting a dual-pathway process. They demonstrated that the reduction pathway highly depends on oxygen coverage: at low oxygen density, oxygen groups are eliminated gradually (mainly hydroxyl and epoxy), in contrast, at high oxygen concentration, abrupt CO/CO_2_ release takes place, resulting in higher defects in the carbon lattice [86]. Building on this, Sengupta et al. (2018) conducted a temperature-dependent analysis from 300 to 450 °C. They showed that ~350 °C is an optimal temperature for annealing, at which a high degree of deoxygenation is obtained while preserving structural integrity. This finding emphasized the practical balance between conductivity restoration and structural stability [87]. Klemeyer et al. (2020) further highlighted the importance of material geometry in the thermal reduction process. They demonstrated that thin GO films reduce faster and more effectively compared to bulk powders due to improved heat transfer and gas diffusion. In contrast, bulk samples need higher temperatures to achieve an equivalent reduction level. This finding underlined the importance of tailoring thermal treatment conditions according to the morphology of the GO starting material [88]. In a recent study, Valentini et al. (2023) have shown the feasibility of thermal reduction (<300 °C) for flexible substrates at low temperature. While this method yielded rGO with lower conductivity compared to those obtained via >1000 °C treatments, it maintained film flexibility, making it highly suitable for portable and flexible electronics [89].

#### 5.2.3. Electrochemical Reduction

Electrochemical reduction has been recognized as a clean, chemical-free, and precisely controllable method to synthesize rGO. Unlike conventional chemical and thermal methods, this method eliminates the need for harmful reagents or high-energy treatments. Instead, an imposed potential directly delivers electrons to GO films or suspensions, which reduce oxygen-containing functional groups as well as partially restore the sp^2^-conjugated carbon framework. Consequently, this method is particularly attractive for sensor fabrication and device integration, since it can be applied directly on electrode surfaces. The process usually involves coating GO onto a conductive substrate, for example, glassy carbon electrodes, metal electrodes or indium tin oxide (ITO), followed by immersion in an electrolyte solution (for instance, phosphate-buffered saline PBS, KCl). A fixed potential or cyclic voltammetry (CV) is then applied, leading to gradual deoxygenation. Reduction level can be adjusted by controlling the applied potential, scan rate, and electrolyte concentration. In most cases, the reduced material is directly suitable for electrochemical applications without further treatment. Wang et al. (2009) reported one of the early studies, in which GO-coated electrodes were reduced electrochemically in electrolyte solution, resulting in rGO with remarkably improved conductivity, which is highly effective for electrochemical sensors [90]. Pei and Cheng (2012) showed its benefits over chemical reduction compared to the chemical process, pointing out that electrochemical reduction methods allow fine control amount of oxygen while reducing chemical waste [91]. Furthermore, previous studies have concentrated on electrolyte engineering. For example, Stankovich et al. (2013) demonstrated that changing the ion composition of the supporting electrolyte markedly influences reduction effectiveness, electrochemical capacity, and long-term stability of rGO films [80].

### 5.3. Graphdiyne

Graphdiyne (GDY), a two-dimensional carbon allotrope characterized by sp-sp^2^ hybridization and evenly distributed acetylenic linkages, is typically synthesized through Glaser–Hay oxidative coupling of terminal alkynes in the presence of copper-based catalysts. Generally, it relies on the deprotection of hexakis[(trimethylsilyl)-ethynyl]benzene (HEB-TMS) to obtain the reactive monomer hexaethynylbenzene (HEB), then subjected to Cu-catalyzed homocoupling to form extended carbon lattices. A typical method involves solution-phase polymerization using organic solvents. For example, deprotected HEB was introduced into a pyridine solution which contained Cu(OAc)_2_ and kept under argon at room temperature for 24 h. The obtained material was purified by consecutive washing with pyridine, DMF, HCl and water and freeze-drying to obtain high-purity GDY [92]. Likewise, CuCl-catalyzed polymerization of HEB-TMS in DMF at 60 °C for 24 h generated a CuO/GDY composite, which was subsequently purified by acid treatment to eliminate copper species, resulting in black-brown GDY nanosheets [93]. This DMF-CuCl approach has been widely popular because of its reproducibility and controllable synthesis. Scale-up methods have been demonstrated in which gram-level synthesis was obtained by the reactivation of several grams of HEB–TMS with CuCl in DMF under the identical conditions [94]. A related CuCl-assisted method involved heating HEB-TMS and CuCl in DMF at 60 °C, 24 h, resulting in CuO/GDY, which, after several solvent washes and HCl treatment, was obtained as pure GDY [95]. Despite these advances in synthesis, 2D-GDY remains rarely employed as a flat-form electrode material in electrochemical pesticide sensing, mainly due to several practical and application-oriented limitations. The synthesis routes are still relatively complex, low-yield, and often suffer from limited reproducibility, particularly when translated to device-level fabrication. Moreover, the diacetylene linkages in GDY are chemically reactive and can undergo degradation under harsh electrochemical environments, such as acidic or alkaline media and repeated redox cycling. In addition, structural defects and poor intersheet contact frequently lead to lower effective electrical conductivity, while the lack of abundant functional groups or heteroatoms limits strong adsorption and selective recognition of pesticide molecules. Consequently, well-established two-dimensional materials, including graphene derivatives and 2D-g-C_3_N_4_, currently offer ultralow detection limits, superior stability, and proven performance in real-sample analysis, leaving limited practical incentive to adopt 2D-GDY for electrochemical pesticide sensing at its present stage of development.

### 5.4. The 2D-g-C_3_N_4_

The 2D-g-C_3_N_4_ is generally produced by pyrolytic condensation of nitrogen-rich, oxygen-deficient compounds containing C-N frameworks, for example, as triazine or heptazine. However, these compounds are often thermally unstable, costly, or reactive, limiting the direct synthesis of single-phase sp^3^ carbon nitrides difficult because of their poor thermodynamic characteristics [96]. Among various approaches, the most widely established method is thermal polycondensation of simple precursors (e.g., cyanamide, dicyandiamide, urea, and melamine). Cyanamide converts into 2D-g-C_3_N_4_ at ~550 °C, evolving NH_3_, whereas NaOH pre-treatment reduces the condensation temperature at ~500 °C, producing a higher surface area and lower aggregation [97]. However, cyanamide is high-cost and reactive, which limits its large-scale use. To address these issues, Xu et al. introduced guanidine hydrochloride, which is a water-soluble precursor and eco-friendly, following a similar condensation route to synthesize g-C_3_N_4_ at 550 °C in Ar [98]. Another low-cost precursor is urea, pyrolysis between 450 and 600 °C to generate 2D-g-C_3_N_4_ with its tunable surface areas (12–83 m^2^/g) varying with calcination temperature [99]. Bulk g-C_3_N_4_ produced by direct condensation typically exhibits very low surface area (~10 m^2^ g^−1^), limiting catalytic efficiency [100]. To overcome these issues, template strategies have been widely employed. Hard templates, for example, SBA-15 or silica, direct the formation of porous structures. On the other hand, surfactants or ionic liquids generate soft templates, such as the incorporation of Fe-g-C_3_N_4_ onto SBA-15, increased surface area (from 8 to 506 m^2^ g^−1^) and improved photocatalytic benzene oxidation [101]. Similarly, ordered mesoporous SBA-15 replicas produced well-defined, highly porous g-C_3_N_4_ [102]. Nevertheless, template removal steps may introduce impurities and complicate the synthesis process. Recent studies have optimized melamine-based methods, such as direct pyrolysis at 600 °C generate bulk g-C_3_N_4_. Then, post-annealing at 500–550 °C for varying durations fine-tunes porosity, crystallinity, and surface activity. Beyond bulk preparation, exfoliation strategies can now enable to formation of a nanosheet [101]. A recent 2023 study outlined multiple exfoliation approaches to enhance the properties of melamine-based g-C_3_N_4_. The one-step thermal route generates bulk g-C_3_N_4_ through calcining melamine, whereas a three-step alkali-assisted strategy delivers edge defects by KOH treatment, resulting in highly reactive nanosheets. Ultrasonic exfoliation in ethanol-water gives an easy, environment-friendly route to generate nanosheets, though precise size control remained a challenge. Thermal exfoliation through re-calcination modestly improved surface area, while chemical exfoliation with H_2_SO_4_ produced highly porous, defective frameworks, albeit requiring careful concerns and neutralization [102]. These methods produce thinner g-C_3_N_4_ nanosheets with high surface areas, adjustable band structures, and enhanced catalytic performance.

## 6. Synthesis Strategies and Their Influence on Electrochemical Sensing Performance

The synthesis procedure of 2D carbon-based materials greatly influences their electrochemical sensing capabilities by regulating essential material properties such as defect density, surface functional groups, conductivity, and active site availability. This section focuses on how various synthesis methodologies affect sensor-relevant features that are essential for pesticide detection rather than just preparation techniques. Commonly employed chemical and electrochemical exfoliation techniques for graphene derivatives generate structural flaws and oxygen-containing functional groups that improve analyte adsorption and enable hydrogen-bonding and π–π interactions with pesticide molecules. However, excessive defects could impair electrical conductivity, necessitating post-treatment or optimization techniques. On the other hand, controlled functionalization and heteroatom doping are made possible by hydrothermal and solvothermal synthesis methods, which enhance charge-transfer kinetics and electrocatalytic activity, especially when metal nanoparticles or heterostructures are included. Thermal polymerization of nitrogen-rich precursors for g-C_3_N_4_ produces a stable framework with numerous nitrogen functionalities that facilitate specific interactions with carbamate and organophosphate insecticides. However, in order to improve electron transport, its very low inherent conductivity requires composite synthesis with graphene or carbon nanotubes. Overall, maximizing electrochemical sensitivity, selectivity, and repeatability in pesticide sensing applications requires adjusting synthesis conditions to balance conductivity, surface chemistry, and defect density.

## 7. Recent Advances in Electrochemical Detection of Pesticides Using 2D Carbon Materials

Pesticides pose a major threat to both human health and the environment, with excessive residues in agricultural products severely compromising food safety. As a result, the sensitive and reliable detection of pesticide residues in vegetables, fruits, and water bodies is essential for ensuring food security and environmental protection. In recent years, 2D carbon materials, including rGO and g-C_3_N_4_, have attracted considerable attention for electrochemical pesticide sensing owing to their large surface area, excellent electrical conductivity, abundant surface functional groups, and strong affinity toward organic contaminants.

### 7.1. Two-Dimensional Carbon Derivative Aptamer-Based Sensing

**Figure 3:**
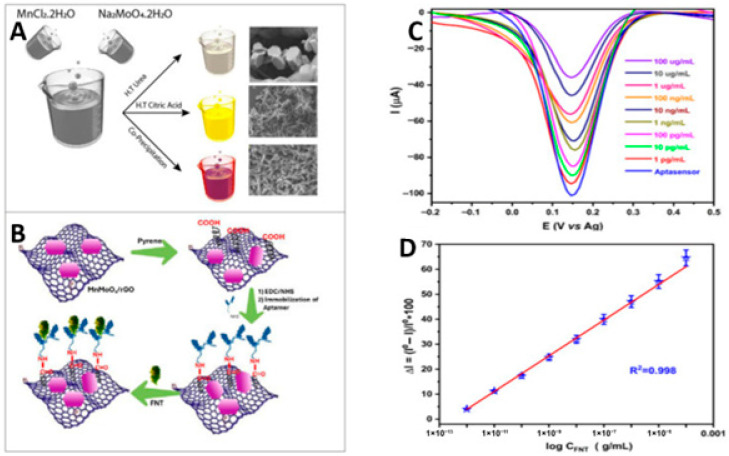
(**A**) Preparation of MnMO_4_/rGO nanocomposite via hydrothermal and co-precipitation techniques. (**B**) Stepwise fabrication of the aptasensor designed for FNT recognition. (**C**) DPV responses of the modified aptasensor (MnMoO_4_/rGO-2:1/pyrene/EDC-NHS/Aptamer-modified electrode) after exposure to different FNT concentrations. (**D**) Corresponding calibration plot for FNT detection (curve of analytical response versus FNT concentration) (Ref. [103]).

Beyond catalytic enhancement, the integration of molecular recognition elements, particularly aptamers, with 2D carbon materials has enabled highly selective pesticide sensing. For example, MnMoO_4_/rGO nanocomposites were successfully employed in an aptamer-based electrochemical biosensor for fenitrothion (FNT) detection [103]. As illustrated in Figure 3A,B, the optimized MnMoO_4_:rGO (2:1) nanocomposite synthesized via a citric-acid-assisted hydrothermal route exhibited a planar hexagonal morphology with superior electrochemical properties. The corresponding DPV responses (Figure 3C) revealed concentration-dependent signal suppression upon FNT binding, while the calibration plot (Figure 3D) demonstrated an ultralow detection limit with excellent linearity. The strong interfacial coupling between MnMoO_4_ and rGO enabled high selectivity against structurally similar pesticides and reliable performance in wastewater, tap water, and rice extract matrices.

**Figure 4:**
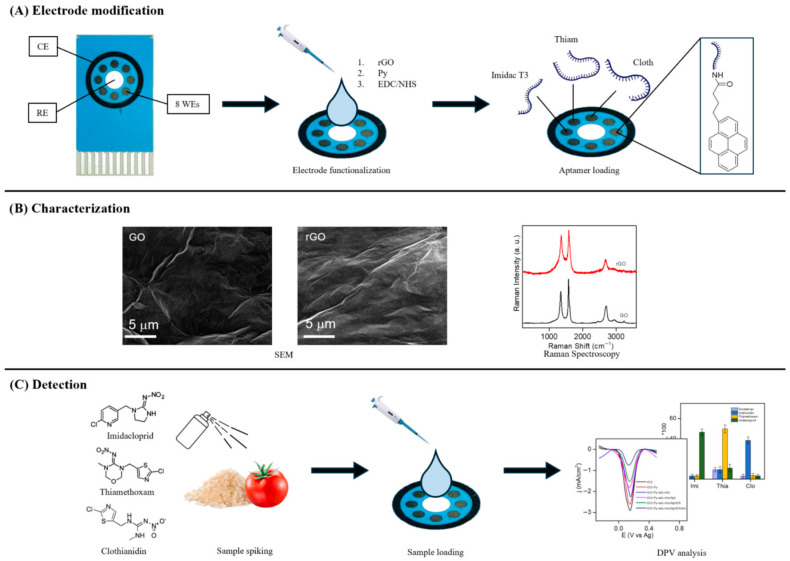
Schematic diagram of the proposed multiplexed aptasensor (Ref. [104]).

In another study, a 2D-rGO-based electrochemical aptasensor was developed by Asma Zaid Al Menhail et al. [104] to detect three hazardous neonicotinoid pesticides: imidacloprid, thiamethoxam, and clothianidin. The 2D-rGO surface was then functionalized with 1-pyrenebutyric acid (py), followed by carbodiimide hydrochloride/N-hydroxy succinimide activation to enable covalent bonding with amine-labelled aptamers (Figure 4). The development of the aptasensor was eventually completed by immobilizing aptamers specific to imidacloprid, thiamethoxam, and clothianidin and blocking any unreacted sites with ethanolamine. Tomato and rice samples were homogenized, extracted with water and acetonitrile, vortexed, centrifuged, evaporated, and then reconstituted in binding buffer. The extracts were filtered and diluted 1:10 before being spiked with known concentrations (10, 50, and 100 ng mL^−1^) of imidacloprid, thiamethoxam, and clothianidin. The spiked extracts were analyzed using the fabricated multiplexed aptasensor. DPV was used to record the current response of each aptamer-functionalized electrode upon exposure to its specific neonicotinoid target. The aptasensor exhibited excellent selectivity and high sensitivity, achieving a detection limit as low as 0.01 ng mL^−1^ across a broad linear range of 0.01–100 ng mL^−1^. The proposed method offers a low-cost, portable, and reliable tool for on-site pesticide detection, thereby contributing to better environmental protection and improved food safety.

### 7.2. Two-Dimensional-Carbon Derivatives with Metal Oxide-Based Sensing

Hybridization of redox-active metal oxides with 2D carbon materials has proven to be an effective strategy for enhancing electrochemical sensing performance. In such architectures, the 2D carbon component serves as a conductive scaffold that facilitates rapid electron transfer, suppresses nanoparticle aggregation, and enhances analyte adsorption through π-π interactions. In that connection, Vinitha M et al. [105] provided a schematic preparation of a metal oxide with 2D carbon nanocomposite GdPO_4_/RGO. To synthesize a nanocomposite, Gd(NO_3_)_3_⋅6H_2_O was taken in deionized water and stirred continuously for 10 min. Citric acid and NaH_2_PO_4_ were then added one after the other. After adding the appropriate amount of 2D-GO, the mixture was continuously stirred for an hour. The entire mixture was then transferred to a Teflon autoclave, which was maintained at 180 °C temperature for 12 h. Thereafter, to remove impurities in the GdPO_4_/RGO, several washes with DI water and ethanol were carried out. Furthermore, EIS and CV studies of bare GCE and modified GCE with GdPO_4_/RGO, which reveal that the bare GCE have limited electron transfer, resulting in a high R_ct_ (886 Ω) and the fabricated GdPO_4_/RGO/GCE exhibited a remarkably low Rct (101 Ω). The DPV curve recorded on the GdPO_4_/RGO/GCE, exhibiting a rapid and sensitive response to FNT additions. Distinct and well-resolved reduction peaks of FNT were observed with broad linear ranges. The reproducibility and spiked real samples, such as river and tap water, were analyzed, demonstrating that the GdPO_4_/RGO/GCE holds potential and is significant for applications in the electrochemical sensing of agricultural pollutants.

Graphitic carbon nitride (g-C_3_N_4_) has emerged as a promising 2D platform for electrochemical sensing owing to its chemical stability, nitrogen-rich framework, and ability to form heterojunctions with metal oxides. Aravind Radha et al. [106] developed a sensor, CaZrO_3_@g-C_3_N_4_ modified glassy carbon electrode (CaZrO_3_@g-C_3_N_4_/GCE) for the detection of diethofencarb (DFC). Firstly, they were synthesized CaZrO_3_ by mixing Ca(NO_3_)_2_ and ZrOCl_2_ in 0.07 L of DI water and then adding KOH solution dropwise while stirring. The complete homogeneous solution was transferred to an autoclave and subjected to maintain temperature up to 100 °C for 24 h. The resulting product, known as CaZrO_3_, was calcined for 3 h at a temperature of around 1000 °C. CaZrO_3_ and g-C_3_N_4_ were combined in a 2:1 ratio and treated under ultrasonication. The resulting final composite (CaZrO_3_@g-C_3_N_4_) was washed and dried at 80 °C. EIS study of CaZrO_3_@g-C_3_N_4_ had higher conductivity and better electron transport performance, as evidenced by a significantly lower Rct (53.4 Ω cm^2^) than bare GCE (1085 Ω cm^2^). The CaZrO_3_@g-C_3_N_4_ electrode electrochemical activity was examined via the CV method. CV curve explores the high electrocatalytic activity of CaZrO_3_@g-C_3_N_4_@ observed by the high Ipa = 20.70 μA (Epa = 0.37 V) and low performance of bare GCE Ipa = 7.01 μA (Epa = 0.41 V). Furthermore, the influence of pH on DFC oxidation revealed optimal performance at neutral pH. DPV measurements demonstrated a wide linear range and low detection limit, with excellent selectivity, repeatability, and reproducibility in real food samples.

To detect malathion in vegetable samples, Waribam S. D. et al. [107] constructed a g-C_3_N_4_-supported CuO-derived biochar (B-CuO/g-C_3_N_4_). Initially, 10 g of melamine monomer was heated to 550 °C to produce a pale-yellow g-C_3_N_4_. Then, the CuO material was synthesized by combining 0.5 g of CuCl_2_·2H_2_O with 0.05 g of urea, followed by the addition of NH_4_OH and sonication. After that, the resulting mixture was then subjected to hydrothermal treatment for 12 h at 150 °C. Finally, the B-CuO/g-C_3_N_4_ composite was then mechanically blended with biochar and heated to 600 °C for 4 h under an inert nitrogen atmosphere. The electrocatalytic performance of B-CuO/g-C_3_N_4_/GCE was analyzed, revealing a lower peak potential separation (ΔEp = 0.324 V) compared to g-C_3_N_4_/GCE, which exhibited a higher peak separation (ΔEp = 0.366 V). Square wave anodic stripping voltammetry (SWASV) analysis showed that the malathion peak current increased proportionally with concentration, exhibiting good linearity and a low detection limit. The reproducibility of the SWASV measurement was experimentally assessed by modifying the electrode 5 times under identical working conditions, with an RSD of approximately 9.69 ± 0.32% (*n* = 5), demonstrating sensor accuracy. To assess the practical feasibility, the sensor was applied for the detection of malathion in tomato and apple extracts, yielding recovery percentages ranging from 87.64 to 120.59%.

In an investigation by Sladana Durdic et al. [108], they developed Co-doped CeO_2_ nanoparticles decorated on the g-C_3_N_4_ surface, which was applied to find out fenitrothion (FNT) in real samples. The authors first synthesized g-C_3_N_4_ by melamine polymerization at 550 °C. For nanoparticle preparation via the hydrothermal method, CoCl_2_·6H_2_O and Ce(NO_3_)_3_·6H_2_O were dissolved in 100 mL distilled water with 3% NH_3_(aq) added dropwise under stirring for 1 h, then combined with a 50 mL g-C_3_N_4_ suspension (0.03 g mL^−1^) and subjected to magnetic stirring followed by 2 h of sonication [108]. CV analysis of GCp/g-C_3_N_4_@Co-doped CeO_2_ revealed a superior Ipa compared to other modified and bare electrodes. EIS study, GCp/g-C_3_N_4_@Co-doped CeO_2_ demonstrated superior electron transport capability, with a significantly lower electron-transfer resistance (Rct) (2874 Ω) compared to bare GCE (6081 Ω). The bare GCp electrode exhibited poor electrochemical performance, with a ΔE_p_ of 531 mV and low intensity peak currents (Ipa = 34.6 μA, Ipc = −28.8 μA). On the other hand, the GCp/g-C_3_N_4_@Co-doped CeO_2_ sensor showed ΔE_p_ of 252 mV, and the superior current intensity of both Ipa and Ipc peaks were 42.6 μA and −38.4 μA, respectively. SWV responses demonstrate that the electrode GCp/g-C_3_N_4_@Co-doped CeO_2_ successfully detects the FNT pesticide in an extensive concentration range of 0.01–13.7 μM, and determined LOQ and LOD were 0.0097 μM and 0.0032 μM, respectively. The suggested method’s RSD for FNT concentrations of 0.08 μM, 0.5 μM, and 4.5 μM were 2.16%, 0.83%, and 0.85% respectively. Additionally, the standard addition method was used for effective sensing of FNT in real samples, such as apple and tap water. These studies collectively demonstrate that synergistic interactions between metal oxides and 2D carbon materials, rather than the intrinsic activity of individual components, govern sensor performance.

### 7.3. Two-Dimensional-Carbon Derivatives with Molecularly Imprinted Polymer-Based Sensing

Molecularly imprinted polymers (MIPs) integrated with 2D-Carbon derivatives provide outstanding electrochemical benefits for pesticide sensing, primarily due to their synergistic effects on sensitivity, selectivity, and practical applicability. The 2D-Carbon with MIPs creates highly specific recognition cavities that match the perfect size, shape, and active functional groups of pesticide molecules, enabling selective targeting even among structurally similar agrochemicals. In this context, a CdMoO_4_/g-C_3_N_4_ based molecularly imprinted electrochemical sensor was developed by Mehmet Lutfi Yola [109] through a combination of material synthesis, electrode fabrication, and analytical application to achieve ultrasensitive carbendazim detection. 2D-g-C_3_N_4_ was first synthesized by thermal condensation of melamine, after which CdMoO_4_ microspheres were uniformly grown on 2D-g-C_3_N_4_ sheets using a one-pot in situ hydrothermal method, forming a heterojunction nanocomposite with enhanced charge-transfer properties. The nanocomposite was drop-cast onto a polished glassy carbon electrode, followed by electropolymerization of pyrrole in the presence of carbendazim as a template to form a molecularly imprinted polymer layer; subsequent template removal generated selective recognition cavities on the electrode surface. Electrochemical characterization revealed that the CdMoO_4_/g-C_3_N_4_ modification significantly reduced charge-transfer resistance and improved electron-transfer kinetics, resulting in a wide linear detection range 1.0 × 10^−5^–1.0 × 10^−3^ µM and an ultralow detection limit of 2.5 × 10^−6^ µM. The sensor exhibited excellent selectivity against interfering pesticides, high stability, reproducibility, and reusability, and was successfully applied to real apple and orange juice samples with recoveries close to 100%, confirming minimal matrix effects. Hence, the synergistic integration of CdMoO_4_ with 2D-g-C_3_N_4_ and the molecular imprinting strategy enabled the construction of a robust, sensitive, and selective electrochemical platform, proving strong potential for practical pesticide monitoring and food safety applications.

Similarly, Ayman H. Kamel et al. [110] synthesized 2D-rGO with MIPs using an emulsion photopolymerization method with imidacloprid as the template, methacrylic acid as the functional monomer, ethylene glycol dimethacrylate as the cross-linker, and benzoyl peroxide as the initiator towards the detection of imidacloprid. Soxhlet extraction was then used to remove the template and create specific recognition cavities, and a non-imprinted polymers were prepared for comparison. A glassy carbon electrode was first modified with 2D-rGO to serve as a solid-contact ion-to-electron transducer, and then a PVC-based sensing membrane containing the MIP beads, plasticizer, and ionic additive was cast onto the 2D-rGO layer to fabricate an all-solid-state potentiometric sensor. The incorporation of 2D-rGO significantly enhanced potential stability, minimized water-layer formation, reduced signal drift, and improved durability, while the MIP layer provided high molecular selectivity toward imidacloprid. The resulting sensor exhibited a wide linear detection range from 0.5 to 1000 µM, and a low detection limit of 0.2 µM. The sensor demonstrated strong selectivity against structurally related neonicotinoids and common interferents, as well as high repeatability and long-term stability. Furthermore, the sensor was successfully utilized to the direct measurement of imidacloprid in commercial pesticide formulations, with recoveries ranging from 94.5% to 106.4%, demonstrating good agreement with HPLC results. A synergistic combination of MIP-based molecular recognition and 2D-rGO-based solid-contact transduction resulted in the development of a cost-effective, sensitive, selective, and stable potentiometric sensing platform appropriate for routine agro-industrial pesticide monitoring and quality control.

### 7.4. Two-Dimensional-Carbon Derivatives with Enzyme-Based Biosensing

Enzyme-based biosensing platforms integrated with 2D-carbon derivatives offer significant electrochemical benefits for pesticide detection. Enzymes such as acetylcholinesterase, organophosphorus hydrolase, and laccase exhibit high biochemical specificity toward pesticides. In contrast, 2D-Carbon derivatives serve as excellent conductive matrices with large electroactive surface areas, promoting rapid electron transfer between the enzyme active sites and the electrode. This results in enhanced current response and lower detection limits. Especially in 2D-g-C_3_N_4_, being nitrogen-rich semiconducting materials, supply abundant surface functional groups that facilitate stable enzyme immobilization through electrostatic interactions and hydrogen bonding, preserve enzymatic activity, and enable strong interaction of pesticide molecules.

Kai Zhang et al. [111] synthesized reduced graphene oxide-chitosan (rGO-CHI) by chemically reducing graphene oxide in an acidic chitosan medium with ascorbic acid to obtain a conductive and biocompatible composite, onto which methamidophos was immobilized as a recognition molecule due to its strong binding affinity toward AChE. A polished glassy carbon electrode was modified by drop-casting the rGO-CHI composite, followed by methamidophos immobilization and bovine serum albumin blocking to prevent nonspecific adsorption, resulting in an electrochemical biosensor based on the AChE inhibition principle. The addition of rGO-CHI greatly improved electron transfer, but the interaction between AChE and organophosphorus pesticides increased charge-transfer resistance, resulting in sensitive current fluctuations that were measured by DPV. The biosensor demonstrated outstanding electrochemical performance, with low detection limits ranging from 0.05 to 0.52 ng mL^−1^, remarkable reproducibility and stability, and the ability to detect various organophosphorus and carbamate pesticides across a wide concentration range. Therefore, the synergistic combination of rGO conductivity, chitosan biocompatibility, and methamidophos-mediated AChE inhibition enabled the development of a highly sensitive, stable, and broad-spectrum electrochemical biosensing platform suitable for rapid and practical pesticide monitoring in food safety applications.

Similarly, Sehrish Bilal et al. [112] prepared a NiCr_2_O_4_/g-C_3_N_4_-based electrochemical AChE biosensor heterostructured composite through an in situ chemical reaction. NiCr_2_O_4_ particles were uniformly deposited onto g-C_3_N_4_ sheets, yielding a high-surface area, conductive, and biocompatible transducer material. The composite was immobilized onto pencil graphite electrodes, functionalized with carboxyl groups, and activated using EDC/NHS chemistry to enable covalent attachment of AChE enzymes extracted from three insect species (Apis mellifera, Tribolium castaneum, and Zootermopsis nevadensis). Electrochemical characterization confirmed enhanced electron-transfer kinetics after composite modification and successful enzyme immobilization, while the resulting bioelectrodes exhibited strong electrocatalytic oxidation of thiocholine and sensitive inhibition responses toward malathion. The biosensors showed species-dependent analytical performance with wide linear ranges and nanomolar detection limits, along with good stability, reproducibility, and selectivity. Practical applicability was demonstrated through the successful detection of malathion in spiked wheat flour samples using the Apis mellifera AChE-based biosensor, which achieved high recovery values comparable to HPLC analysis. The synergistic combination of NiCr_2_O_4_ and g-C_3_N_4_ provided an efficient enzyme-immobilization matrix and signal-amplifying platform. This highlights the strong potential of such metal oxide g-C_3_N_4_ composites for food safety monitoring, sensitive pesticide toxicity assessment, and environmental analysis.

### 7.5. Structure–Property–Performance Relationships

Across the reported studies, clear structure–property–performance relationships can be identified. Extended 2D architecture gives abundant electroactive sites and facilitates rapid electron transport, leading to lower LOD. Heterojunction formation between 2D carbons and metal oxides reduces interfacial resistance and enhances signal stability. Metal doping and composite engineering introduce additional redox-active sites, improving sensitivity and widening the linear detection range. Meanwhile, surface modification with aptamers enables highly selective biosensing platforms with least interference effects.

### 7.6. Practical Applicability and Outlook

A key advantage of 2D carbon-based electrochemical sensors lies in their demonstrated applicability to real-world samples. Reliable detection of pesticide residues has been achieved in wastewater, tap and river water, and food matrices such as tomato, rice, apple, grape, and leafy vegetables, with acceptable recovery values and low relative standard deviation, as summarized in previous publications [99,100,101,102,103,104,105,106,107]. The malathion sensor based on B-CuO/g- C_3_N_4_ further highlights this applicability, where the synthesis route, electrochemical behaviour, and SWASV responses collectively demonstrate high sensitivity and real-sample feasibility [107].

Similarly, 2D carbon materials have become appealing options for direct electrochemical sensing and reliable templates for the production of 2D carbon-based electrochemical sensing materials. These materials have the potential to improve the sensitivity of electroanalytical procedures for sensing pharmaceuticals, environmental contaminants, and electrochemical energy storage systems. Since the existence of these hazardous pesticide compounds poses exposure and potential bioaccumulation dangers in organisms, it is crucial to develop innovative ways for monitoring and controlling their concentrations. As a result, establishing a sensitive and efficient pesticide detection method is crucial for environmental monitoring and improvement (recent improvements were noted in Table 1). Furthermore, platforms such as g-C_3_N_4_ nanosheet modified electrodes have made it possible to detect different residues in environmental and food matrices quickly, reliably, and without interference, which supports more efficient environmental monitoring and food safety (Table 2). The exceptional analytical capability demonstrated by these 2D carbon-driven electrochemical sensors is significantly relevant to today’s environmental and food safety assessment. The intrinsic advantages of 2D materials, particularly their large surface area, abundant functional active sites, rapid charge transport, and customizable surface chemistry support the fabrication of sensors with remarkably low LOD, often reaching the ng mL^−1^ level [104]. Such sensitivity is essential for monitoring pesticide residues in water as well as agricultural products, since regulatory policies limits require trace-level detection to ensure public and environmental health. Several recent reviews have emphasized that graphene, rGO, g-C_3_N_4_, and related 2D materials possess remarkable electrocatalytic performance and fast electron-transfer kinetics, making them ideal for monitoring pollutants such as neonicotinoids, organophosphates, carbamates, heavy metals, and antibiotics in a wide range of environmental matrices [113,114,115]. Additionally, the use of electrochemical techniques such as differential pulse voltammetry (DPV), square wave voltammetry (SWV), and square wave anodic stripping voltammetry (SWASV) facilitates the development of cost-effective, miniaturized, and portable sensing devices that demand minimal instrumentation is an essential feature for real-time and field-deployable analysis. The successful application of these 2D carbon-based sensors in complex real-world matrices, including wastewater, tomato, rice, apple, and tap/river water extracts (Refs. [97,99,100,102]), further demonstrates their stability, excellent recovery rates, and low RSD values. These findings confirm their ability to function efficiently without requiring extensive laboratory-based preprocessing. Overall, the ease of portability, analytical reliability, and strong real-sample compatibility of 2D-carbon-based sensors make them as powerful tools for quick hazard assessment, environmental monitoring, and ensuring food safety, thus significantly contributing to global health as well as environmental protection efforts.

## 8. Conclusions and Future Perspective

The determination of pesticides is essential because their consumption by humans creates serious health issues, such as neurological disorders and cancer, and also pollutes soil, water, and air. Two-dimensional carbon materials, including graphene, rGO, graphdiyne, and g-C_3_N_4_, as well as their mixed nanocomposites, have shown remarkable electrocatalytic properties in the electrochemical detection of hazardous contaminants, biomolecules, and various heavy metals. The significant sensing ability of 2D carbon materials is due to their high sensitivity, selectivity, vast surface area, adjustable defects, excellent electrical conductivity, and rapid analysis. Hence, these materials meet the demands in food safety and environmental monitoring applications. In addition, 2D carbon-based materials also show remarkable detecting efficiency towards pesticides, with a dynamic range and low limits of detection supported by their distinctive physicochemical characteristics.

Here, we thoroughly reviewed the recent developments in the electrochemical determination of pesticides using 2D-carbon materials as an electrocatalyst. We also discussed the toxicity of various pesticides. The synthesis, properties, and advantages of 2D carbon materials are discussed in detail. The fabrication of various electrochemical strategies towards pesticide detection is elaborately addressed. The electrochemical sensing methodology, the versatility of 2D carbon materials, selectivity, and real sample analysis of pesticides are properly discussed. Although electrochemical sensors have demonstrated a great deal of promise for pesticide detection, their in situ environmental application is still difficult. Analytical dependability is generally limited by complex sample matrices in natural streams and agricultural runoff, which frequently result in interference, electrode fouling, and decreased selectivity. Furthermore, in variable field circumstances like temperature, pH, and ionic strength, many sensors have poor long-term stability and signal drift. Even though reports of ultra-low detection limits are common, on-site applications still struggle to strike a balance between high sensitivity and real-time monitoring. Large-scale implementation is further hindered by problems with reproducibility and calibration, and continuous monitoring is hampered by limited integration with portable and wireless systems. Therefore, the development of strong antifouling electrode materials, highly selective recognition techniques, standardized fabrication procedures, and clever data-processing methods should be the main emphasis of future research. Transitioning electrochemical sensors from lab research to useful in situ pesticide monitoring will require a focus on sensor miniaturization, field validation, and comparison with traditional analytical methods.

Future research should focus on the simultaneous detection of multiple pesticides using a single electrochemical technology. It will be a challenging task to find a remarkable 2D carbon-based electrocatalyst that can detect multiple target molecules simultaneously. Furthermore, it would be essential to identify the effect of environmental conditions, including temperature, pH, and humidity, on the reliability and accuracy of the sensors. To create reliable, field-ready sensors, it is important to study functionalization techniques, composite creation, and device engineering. In addition, enhancing selectivity, developing economical and sustainable synthesis techniques, integrating sensors with digital platforms for real-time monitoring, and encouraging extensive testing in environmental and agricultural settings are some future prospects.

## Figures and Tables

**Table 1:** Recent applications of 2D carbon material-based electrochemical sensors for pesticide detection.

2D-Carbon with Composite	Targeted Pesticide/Class	Method	LOD	Linear Range	Real Sample	Reference
GO@Ce-doped TiO_2_	methyl parathion	DPV	0.0016 μM	0.002–48.327 μM	apple and tomato	[116]
Zn-Ni-P-S/GO/GCE	paraoxon ethyl	AMP	0.035 μM	1–200 μM	agriculture wastewater	[117]
AgNPs@GO/IL@SPCE	methyl parathion	SWV	9 μM	25–200,000 μM	ground water and surface water	[118]
CP5-rGO/GCE	methyl parathion	DPV	0.0003 μM	0.001–150 μM	soil and tap water	[119]
CeO_2_-CuO/rGO	methyl parathion	DPV	0.00179 μM	0.0038–0.152 μM	mineral, drinking, and tap water samples	[120]
FeVO_4_/RGO	methyl parathion	AMP	0.00070 μM	0.001–20 μM	green beans	[121]
RGO-CHI	parathion dichlorvos methamidophos trichlorfon dimethoate fenthion methomyl chlorpyrifos omethoate diazinon	DPV	0.05–0.52 ng mL^−1^	0–1500 ng mL^−1^	NA	[111]
Pd@NrGO	methyl parathion	SWASV	1.067 ng mL^−1^ 122 ng mL^−1^	1.8–10 ng mL^−1^ 50–1000 ng mL^−1^	river water, onion, agricultural run-off, cabbage, lettuce leaves and tap water	[122]
SnS_2_/NS-RGO	methyl parathion	CV	0.00017 μM	0.001–176 μM	xindian river and black grapes	[123]
YFO-rGO/RDE	carbofuran	i-t	0.018 μM	0.029–303.5 μM	carrots, cucumber, cabbage, spinach, tomato and potato.	[37]
NiO/MoS_2_/Rgo	methyl parathion	DPV	1.1 ng mL^−1^	10–10,000 ng mL^−1^	apple juice	[124]
N-HG_50_/GCE	methyl parathion	DPV	0.013 nM	1 ng mL–150 μg mL^−1^	apple, grape, cucumber and river water	[125]
Au@rGO/CuO/GCE	methyl parathion	SWV	0.045 μM	0.4–39.0 μM	Dhaleswari river	[126]
AuNPs@GO/IL@SPCE	pirimicarb	SWV	4.49 μM	50–1500 μM	groundwater and surface water	[127]
ZrO_2_/rGO	methyl parathion	GECT	10 pg mL^−1^	1 × 10^−5^–10 μg L^−1^	Chinese cabbages	[128]
CeO_2_/NiO/GO	carbofuran	DPV	0.82 μM	5–150 μM	potato	[129]
CPE-rGO	carbofuran	DPV	0.0023 μM	0.03–0.8 μM	drinking water, lettuce leaves, orange juice, wastewater	[130]
Cu-rGO	imidacloprid	CV	0.003247 μM	NA	soil sample from paddy field	[131]
GO/Au NPs/β-CD	imidacloprid	DPV	0.000133 μM	5 × 10^−4^–0.3 μM	Chinese cabbage, banana and mango	[132]
GCE/rGO/MIP	imidacloprid	Potentiometry	0.8 μM	1–1000 μM	NA	[110]
E-rGO/CDs	imidacloprid	CV	0.02 μM	0.5–40 μM	brown rice	[133]
AgNDs/GNs/GCE	imidacloprid	DPV	0.814 μM	1–100 μM	cucumber	[134]
GCE/rGO/MnPc	imidacloprid	CV	6.5 μM	25–250 μM	honey	[135]
SPCE/GO/AuNPs/P_3_ABA	paraquat	SWV	4.5 × 10^−4^ μM	0.001–100 μM	tap water and natural water	[136]
MnMoO_4_/rGO	fenitrothion	DPV	3.0 × 10^−4^ ng mL^−1^	1.00 × 10^−3^–1.00 × 10^5^ ng mL^−1^	wastewater and rice extract	{103]
GdPO_4_/RGO	fenitrothion	DPV	0.007 μM	0.01–342 μM	river and tap water	[105]
rGO-aptamer	imidacloprid thiamethoxam clothianidin	DPV	6.30 pg mL^−1^ 6.80 pg mL^−1^ 7.10 pg mL^−1^	0.01–100 ng mL^−1^	rice and tomato water	[104]

**Table 2:** Recent applications of g-C_3_N_4_-based electrochemical sensors for pesticide detection.

2D-Carbon with Composite	Targeted Pesticide/Class	Method	LOD	Linear Range	Real Sample	Reference
KL@Ni@g-C_3_N_4_	Cypermethrin	CV	0.026 μg mL^−1^	0.05–0.2 μg mL^−1^	Tap Water	[58]
g-C_3_N_4_@LiCoO_2_	Malathion	DPV	0.00438 μM	0.005–0.12 μM	Lettuce	[57]
GO/g-C_3_N_4_	Methyl parathion carbendazim	SWV	8.4 × 10^−4^ μM 2.0 × 10^−6^ μM	0.08–100 μM 0.01–250 μM	Water and Soil sample	[55]
CdS/g-C_3_N_4_/Sm-BDC	Malathion	DPV	0.0074 μM	0.03–0.15 µM	Cabbage	[137]
B-CuO/g-C_3_N_4_	Malathion	SWASV	1.2 pg mL^−1^	0.18–5.66 pg mL^−1^	Soil, Rice, Water and Fruits	[107]
g-C_3_N_4_@Co-doped CeO_2_	Fenitrothion	SWV	0.0032 μM	0.01–13.70 μM	Tap water and Ethanolic apple extract	[108]
CaZrO_3_@g-C_3_N_4_	Diethofencarb	DPV	0.0018 μM	0.01–230.04 µM	Strawberry, Grapes, Spinach, and Apple	[106]
MIP/CdMoO_4_/g- C_3_N_4_	Carbendazim	CV	2.5 × 10^−6^ µM	1.0 × 10^−5^–1.0 × 10^−3^ µM	Fruit juice	[109]
Fe_2_O_3_@g-C_3_N_4_ @MSB	Thiamethoxam	LSV	0.137 µM	0.01–200 µM	Potato, Rice and River water	[138]
g-C_3_N_4_/CPE	Dichlorophen Thymol	SWV	0.012 µM	0.05–100 µM	Soil and Water	[139]
g-C_3_N_4_/EuMoO_4_	Carbendazim	DPV	0.04 µM	50–400 µM	Apple and Tomato	[140]
rGO-g-C_3_N_4_-MnCo_2_O_4_	Chlorpyrifos	DPV	0.32 × 10^−6^ μg mL^−1^	1.5 × 10^−5^–7.0 µg mL^−1^	Groundwater, Tap water and Pomegranate sample	[141]
KL@Ni@S-g-C_3_N_4_	Cypermethrin	CV	0.05 µg mL^−1^	0.1–1.0 µg mL^−1^	Tap water	[54]
NiCr_2_O_4_/g-C_3_N_4_	Malathion	CV	0.0023 µM	0.002–0.10 µM	Wheat flour	[112]
Sr_x_Zn_1-x_O/g-C_3_N_4_	Glyphosate	DPV	1.4 × 10^−4^ µM	0.002–0.1 µM	Lake Sapanca, Tap water and Natural Spring water.	[142]
PTH-g-C_3_N_4_	Carbendazim	DPV	3.7 × 10^−4^ µM	0.1–85 µM	Cherry Wine	[143]
C_3_N_4_-MoS_2_-Au	Pendimethalin	LSV	0.219 µM 0.615 µM 0.479 µM	0–1000 µM 0–500 µM 0–500 µM	Tap water and Distilled water	[144]
g-C_3_N_4_/GO/Fc-TED	Metolcarb	DPV	0.0083 µM	0.045–213 µM	Vegetable Spinach	[145]

## Data Availability

No new data were created or analyzed in this study. Data sharing is not applicable to this article.
